# Healthcare Settings as Amplifiers of Infectious Disease^1^

**DOI:** 10.3201/eid1011.040797_01

**Published:** 2004-11

**Authors:** Linda A. Chiarello, Michael L. Tapper

**Affiliations:** * Centers for Disease Control and Prevention, Atlanta, Georgia, USA;; † Lenox Hill Hospital, New York, New York, USA

**Keywords:** healthcare settings, infectious disease, global outbreaks, SARS

Global outbreaks of severe acute respiratory distress syndrome (SARS) in 2003 demonstrated the potential of healthcare facilities to serve as amplifiers of a new communicable disease. However, healthcare settings can also be amplifiers of multidrug-resistant bacteria and bloodborne viruses.

In the public health and healthcare delivery systems, amplifying forces include weaknesses in communication, coordination, early detection and control of emerging diseases, and oversight of healthcare services. Among healthcare personnel, weaknesses include gaps in infection control knowledge and practice.

SARS was spread globally by relatively few people and amplified by super-spreading events that occurred primarily in healthcare settings. Healthcare personnel were disproportionately affected, accounting for up to 57% of cases in some countries. The combination of increasing infectivity in the later stages of SARS, performance of the aerosol-generating procedures (bronchoscopy, intubation), and clustering of SARS patients further enhanced transmission of the SARS-associated coronavirus.

Systems for early detection and isolation of persons with suspected SARS and public quarantine effectively reduced transmission. Conversely, absence of control measures at initial points of patient encounter, particularly in hospital emergency departments, rendered hospitals particularly vulnerable to SARS transmission.

Unsafe injection and blood donation practices have contributed to the global spread of bloodborne viral diseases. Worldwide, unsafe injections alone are estimated to cause 21,000 cases of hepatitis B, 2,000 cases of hepatitis C, and 260 cases of HIV each year. Countries with limited resources are at a disproportionate risk for adverse injection-related outcomes. While lack of sterile supplies is important, unnecessary injections and poor understanding of infection control principles and practices also contribute to the spread of bloodborne viruses. These last two factors are not unique to the developing world. Four recent outbreaks of hepatitis B and C viruses in patients in ambulatory care facilities in the United States are a reminder that unsafe injections can occur in any healthcare setting. In these outbreaks, a lack of administrative oversight and poor understanding of infection control practices contributed to the contamination of multidose vials or the reuse of injection equipment and transmission of hepatitis B or hepatitis C virus to numerous patients.

In contrast to SARS and bloodborne viruses, the rise and amplification of multidrug-resistant organisms in healthcare settings have been gradual and subtle. These organisms limit treatment options, increase transmission risks for vulnerable patient populations, increase illness and death, prolong the hospital stay, and add to healthcare costs. The rise of these organisms has been most dramatic in U.S. intensive care units, where >50% of *Staphylococcus aureus* isolates are resistant to methicillin (MRSA) and >25% of enterococcal isolates are resistant to vancomycin. Cases of vancomycin-intermediate *S. aureus* and three recent cases of vancomycin-resistant *S. aureus*, both in outpatient settings, attest to the potential for amplification of these organisms in healthcare settings. Gram-negative organisms resistant to extended-spectrum β-lactamases present similar concerns and have been associated with numerous outbreaks in healthcare facilities.

The problem of multidrug-resistant organisms is multifaceted. While colonized and infected patients constitute the major reservoir for dissemination of these organisms, inappropriate use or overuse of antimicrobial agents contributes to acquiring and expressing resistance genes. Healthcare settings become breeding grounds of additional resistance and distribution centers for amplification of multidrug-resistant organisms to other healthcare settings and the community.

The notion that our healthcare settings contribute to the amplification of infectious disease contradicts our expectations. Usually, healthcare systems work well, and quality healthcare is delivered safely and efficiently. Nonetheless, there are gaps in infrastructure, knowledge, and practice that can open the door to disease outbreaks.

